# Synthesis and characterization of vertically standing MoS_2_ nanosheets

**DOI:** 10.1038/srep21171

**Published:** 2016-02-18

**Authors:** Han Li, Huaqiang Wu, Shuoguo Yuan, He Qian

**Affiliations:** 1 Institute of Microelectronics, Tsinghua University, Beijing, China; 2 Tsinghua National Laboratory for Information Science and Technology (TNList), Beijing, China; 3 Department of Applied Physics, The Hong Kong Polytechnic University, Hong Kong, China

## Abstract

Molybdenum disulfide (MoS_2_) has been attracting much attentions due to its excellent electrical and optical properties. We report here the synthesis of large-scale and uniform MoS_2_ nanosheets with vertically standing morphology using chemical vapor deposition method. TEM observations clearly reveal the growth mechanism of these vertical structures. It is suggested that the vertical structures are caused by the compression and extrusion between MoS_2_ islands. More importantly, the vertical morphology of two dimensional (2D) materials hold many promising potential applications. We demonstrate here the as-synthesized vertically standing MoS_2_ nanosheets could be used for hydrogen evolution reaction, where the exchange current density is about 70 times of bulk MoS_2_. The field emission performance of vertically standing MoS_2_ were also improved due to the abundantly exposed edges.

Graphene has attracted extensive interests in various research fields since it was obtained through mechanical exfoliation by Novoselov *et al*. in 2004^1^. Due to the distinctive physical properties of one-layer thin 2D materials compared with their bulk counterparts^2^,^3^, layered materials have attracted much attentions, such as transition metal dichalcogenides (TMDCs)^4^, transition metal oxides^5^, boron nitride (BN)^6^, *etc*. Lots of efforts have been made by using 2D materials in the fields of microelectronics^7^,^8^, optoelectronics^9^, sensors^10^ and energy storage^11^,^12^. However, these works have been devoted to utilize 2D materials lying flat on the substrates. Less of attention has been paid on their alternative configuration^13^,^14^,^15^. Amongst these nanometric architectures, vertically standing 2D materials hold great potential in many applications due to their high aspect ratio and extensively exposed edges^16^.

For example, the minimized dimension and vertically aligned morphology of 2D materials consequently enable the fabrication of mini-sized energy storage devices with high capacity and high packing density, such as hydrogen storage devices, batteries and supercapacitors. The exposed edges with dangling bonds are chemical active and may play an important role in many catalytic reactions, such as hydrodesulfurization, hydrogen evolution reaction (HER) etc^17^,^18^,^19^,^20^. J. Shi *et al*.^20^ demonstrate that the HER activity relates closely to the edge sites of MoS_2_ flakes and the basal surfaces are catalytically inert, revealing the importance of exposed edges in catalytic reactions^21^. Furthermore, It has been demonstrated that vertically growth 1D nanotubes/nanowires and 2D nanosheets with atomically thin edges can significantly improve the field emission properties^22^,^23^, making vertically standing 2D materials promising candidates in field emission applications^24^,^25^,^26^.

There have already been lots of works based on the one dimensional (1D) nanowires and nanotubes. And the growth mechanism for vertical 1D nanowires and nanotubes are widely discussed. Normally, nanowires and nanotubes are assumed to be grown at the interface between catalytic and nanowires (nanotubes) via vapor-liquid-solid (VLS) or vapor-solid-solid (VSS) process in 1D growth^27^,^28^. In contrast, the growth mechanisms for vertically standing 2D materials are still vague. L. Jiang *et al*.^24^ demonstrated that a transition from 2D complete films to 3D clusters beyond a critical layer thickness may be caused by the sufficient accumulation of strain energy and the defects of the as-deposited film during vertical graphene growth process. J. Zhao *et al*.^29^ demonstrated that vertically standing graphene could be nucleated from the buffer layer or from the surface of carbon onions. However, there are no clear evidences demonstrating that how these transitions from 2D films to 3D clusters happened. We believe that a clear understanding of the growth mechanism would facilitate the development of vertically standing materials based applications. In addition, the growth mechanism could also promote the process in designing more complex nanometric structures.

In this work, we propose a method to synthesize vertically standing MoS_2_ nanosheets using a conventional chemical vapor deposition (CVD) method. Various characterizations techniques were used to give a deep analysis of those vertically standing MoS_2_ nanosheets. In addition, a possible mechanism is proposed based on the experimental results. Furthermore, the field emission properties and the HER performance of vertically standing MoS_2_ nanosheets were reported.

## Results

### Synthesis and characterization of vertically standing MoS_2_

**Figure 1:**
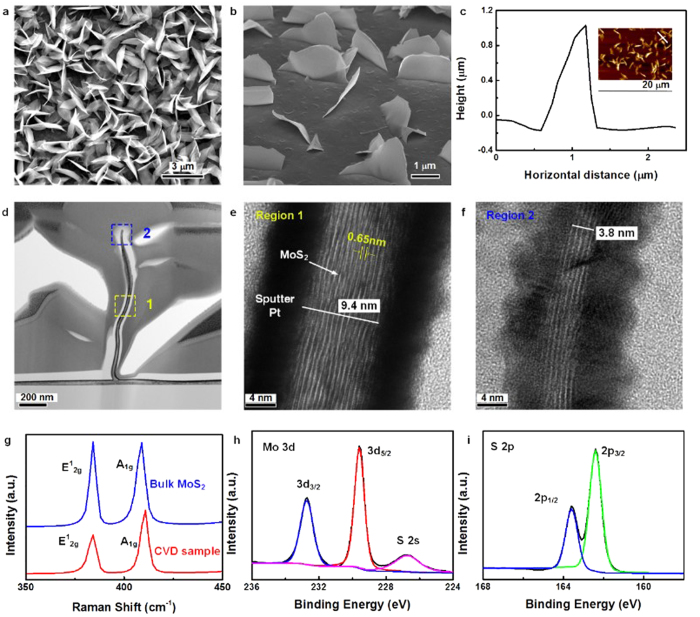
Characterization of as-synthesized MoS_2_ nanosheets. (**a)** Top view FE-SEM image of vertically standing MoS_2_ nanosheets. (**b)** 52.5° tilt view of MoS_2_ nanosheets which clearly show the vertically standing feature. (**c)** AFM height profile of MoS_2_ nanosheets, the insert shows the corresponding AFM image. (**d)** Cross-sectional TEM image of vertically standing MoS_2_ grown on SiO_2_/Si substrate. (**e)** Region1 (yellow block in d): High-magnification cross-sectional TEM image of MoS_2_ layers, clearly shows the layered structure of MoS_2_ nanosheets. (**f)** Region2 (blue block in d): High-magnification cross-sectional TEM images on the top of vertically standing MoS_2_ nanosheets, and nanometric edge with size of several nanometers could be observed. (**g)** Raman spectrum of our sample and single-crystal bulk MoS_2_ obtained by mechanical exfoliation. (**h)** XPS spectra of Mo 3d and S 2s peaks. (**i)** XPS spectra of S 2p peak.

A typical SEM image of the vertically standing MoS_2_ nanosheets grown on SiO_2_/Si substrate is shown in Fig. 1a. It clearly shows that MoS_2_ nanosheets were uniformly grown on the substrate. The 52.5° tilt SEM image (Fig. 1b) demonstrates that the as-grown MoS_2_ nanosheets are nearly perpendicular to the substrate. AFM height profile is shown in Fig. 1c, where the corresponding AFM image is shown in the inset. The nanometric edges of different MoS_2_ layers could be clearly observed in the high magnification SEM image (see Supplementary Fig. S1 online). It is believed that these nanometric protrusions and edges are catalytically active sites which could significantly enhance the catalytic and field emission properties.

To further explore the microstructure and quality of the as-grown MoS_2_ nanosheets, cross-section TEM analysis were carried out. TEM samples were prepared using Focused Ion Beam (FIB) process. The cross-section TEM image (Fig. 1d) shows the general morphologies of a vertically free standing MoS_2_ nanosheets grown on SiO_2_/Si substrate. The synthesized MoS_2_ nanosheet stands nearly perpendicularly upon the substrate, and the height of this MoS_2_ sheet is about 1.1 μm. High resolution TEM images are shown in Fig. 1e,f, corresponding to the region 1 and 2 marked in Fig. 1d respectively, where layered structure of MoS_2_ could be clearly observed. The distance between two MoS_2_ layers was measured to be about 0.65 nm (Fig. 1e), which is consistent with previous reports^7^,^30^. The thickness of MoS_2_ tip was measured to be 3.8 nm, corresponding to 5 layers of MoS_2_. It’s worth to note that the thickness of MoS_2_ nanosheet is non-uniform. The thickness of MoS_2_ increased from 3.8 nm to be 9.4 nm in the middle region of the sheet (Fig. 1e,f), revealing a tapered morphology of MoS_2_ nanosheets. More morphology TEM images could be found in the Supplementary Fig. S2. The same morphology observation was reported in the free-standing vertical graphene by Zhao *et al*.^29^ It’s believed that these pyramid-like shape was caused by the terraces and steps formed during the growth, which also maintain the stability of the vertical structure.

MoS_2_ nanosheets were also characterized using Raman spectroscopy. A typical Raman spectrum of our CVD grown MoS_2_ sample is displayed in Fig. 1g. Two Raman characteristic bands at 410 and 384 cm^−1^ with the full-width-half-maximum (FWHM) values of 5.8 and 5.9 cm^−1^ could be found, corresponding to the out-of–plane A_1g_ and in-plane E^1^_2g_ vibration of MoS_2_ respectively^30^,^31^,^32^. Figure 1g also presents the Raman spectrum of a single-crystal bulk MoS_2_ obtained by mechanical exfoliation as a reference. The similar value of FWHM between CVD samples and pristine MoS_2_ reveals the high quality of CVD grown MoS_2_. Note that the Raman peak corresponding to the out-of-plane mode (A_1g_) is determined by sulfur atoms vibrating along c axis while the in-plane E^1^_2g_ mode are seen to involve motions of molybdenum and sulfur atoms in the basal plane^33^. Thus it can be inferred that there are more exposed edges in the MoS_2_ films grown by CVD than those obtained by mechanical exfoliation. Figure 1h,i display detailed XPS spectrum of Mo and S binding energies. The survey scan is provided in Supplementary Fig. S3. None oxidized Mo or S is found after the examination of Mo and S peaks.

### Growth mechanism for vertically standing MoS_2_ nanosheets

**Figure 2:**
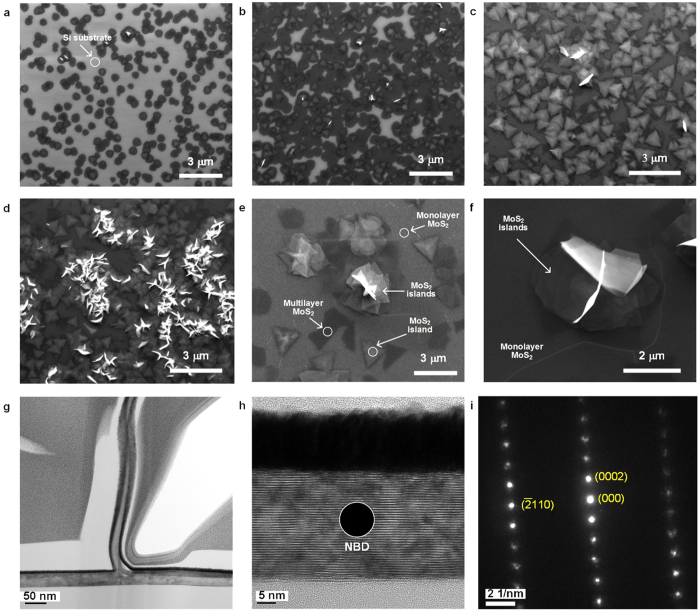
SEM and TEM observation on the formation of vertically standing MoS_2_ nanosheets. **(a–d)** SEM images of the MoS_2_ films grown at the same condition but with different growth time: (**a)** 2 minutes, (**b)** 3 minutes, **(c)** 5 minutes and (**d)** 7 minutes. (**e–f)** SEM observations on the initial stage of the vertical MoS_2_ formation. (**g**) Cross-sectional TEM image of vertically standing MoS_2_. (**h)** HR-TEM image of plane MoS_2_ nanosheets. (**i)** NBD pattern from h (selected area marked by the circle).

To elucidate the growth mechanism of these vertically standing MoS_2_ nanosheets, different samples with varied growth time were prepared and examined. After 2 minutes growth in 750 °C, many speck-like features could be observed (Fig. 2a), and it is believed that those spots are the nucleation sites for horizontal growth of monolayer MoS_2_ films. With growth time continued, most seeds grew into larger domain sizes and some seeds merged into one uniform film, and some triangular MoS_2_ islands were formed on top of MoS_2_ films at the same time (Fig. 2b). More triangle-shaped MoS_2_ islands were formed above MoS_2_ films after 5 minutes growth (Fig. 2c), after that, a transformation from 2D growth to 3D growth started (Fig. 2d). Raman spectroscopy analysis have been applied on those samples with different growth time. As shown in Supplementary Fig. S4, the intensity ratios between A_1g_ and E^1^_2g_ modes of CVD grown MoS_2_ nanosheets samples are higher than that of bulk MoS_2_ samples, revealing a higher density of exposed edges in those CVD grown MoS_2_ nanosheets samples. With growth time increased from 5 minutes to 10 minutes, the ratio between A_1g_ and E^1^_2g_ mode intensity was also increased, suggesting a transformation from 2D to 3D growth.

More SEM observations were performed on the intermediate state of MoS_2_ nanosheets to find out how these vertical MoS_2_ nanosheets are formed. It could be observed in Fig. 2e,f that MoS_2_ seedlings are originated from the aggregation zone of different MoS_2_ islands. Thus, it can be inferred from the SEM observations that the formation of vertical structures were caused by the interaction between different MoS_2_ islands. Cross-section TEM of a vertical MoS_2_ nanosheet (Fig. 2g) exhibits this nanosheets originates from a thick MoS_2_ film at the bottom. High resolution TEM image (Fig. 2h) shows the thick MoS_2_ film has clear layered structure. From the TEM observations, it could be observed that the vertical MoS_2_ nanosheet are originated form the MoS_2_ island layers. Based on above results, it could be inferred that vertical standing MoS_2_ seedlings may originate due to the curling force introduced by the increasing defects and strain energy^34^. Figure 2i display the nanobeam electron diffraction (NBD) pattern from Fig. 2h (selected area marked by the circle), which shows only regularly arranged diffraction spots, corresponding to the hexagonal crystal structure of MoS_2_.

**Figure 3:**
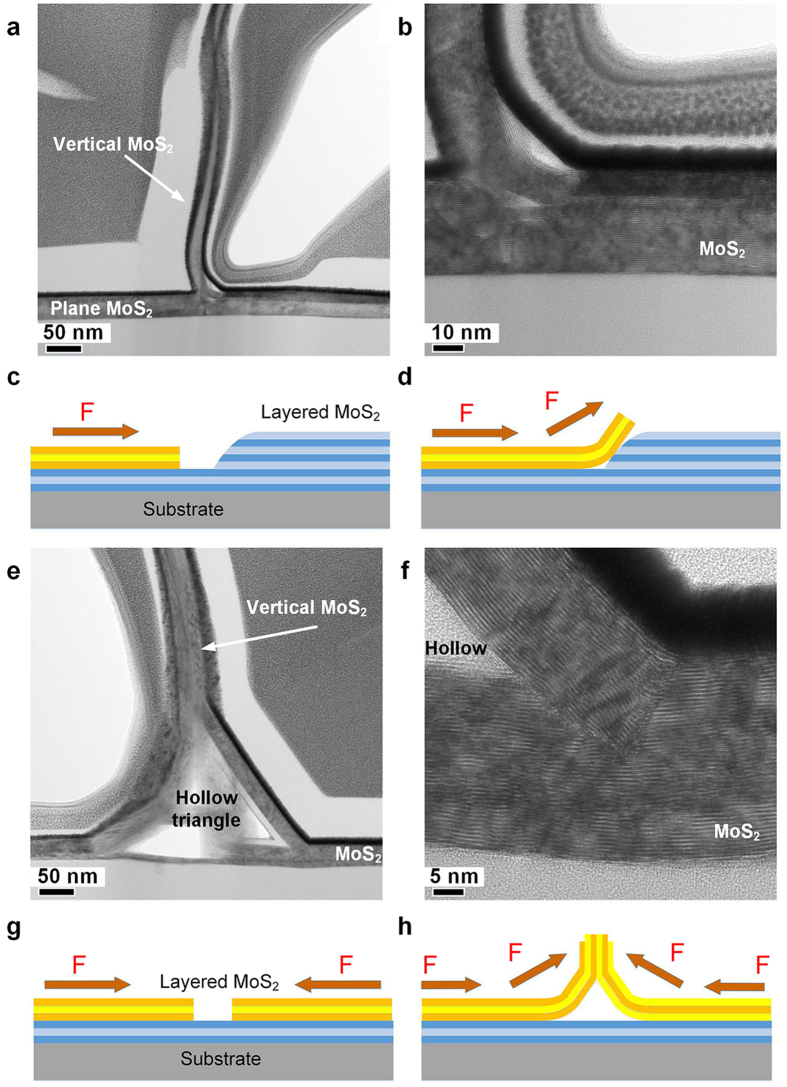
Two types of vertical morphologies and their corresponding structure models. **(a–d)** Type I and their corresponding models. (**e–h)** Type II and their corresponding models.

TEM analyses were performed on the collision or distortion area where the vertically standing MoS_2_ nanosheets nucleated from. Basically, two kinds of vertical morphology and their corresponding base structure were observed (Fig. 3). It can be clearly observed in the TEM images that the vertical MoS_2_ nanosheets were grown from the buffer layer underneath on both cases. The first kind of vertical morphology is shown in Fig. 3a,b. The MoS_2_ nanosheets are originated from the buffer layers and formed by the extension and the curl of the plane MoS_2_ buffer layers. Alternatively, the vertical structure could be originated from the merge of two separated MoS_2_ films (Fig. 3e,f). The formation of vertical morphology may be caused by the collision between two MoS_2_ islands. The same triangular structure could be clearly observed at the intermediate state as shown in Supplementary Fig. S5. In addition, the stable triangle could be observed on the bottom structure on other TEM observations. Energy-dispersive X-ray spectroscopy (EDX) (see Supplementary Fig. S6 online) certify that nearly no chemical elements are existed in the triangle area, suggesting that the empty triangle was formed due to the curl of MoS_2_ films instead of other chemical reaction. We believe that the self-formative triangular area could enhance the stability of the whole vertical structure.

Based on the above observations, a vertical growth mechanism is proposed to explain the formation of vertically standing MoS_2_ nanosheets. It is believed that the intensive compression between different MoS_2_ islands cause the collision and slide of MoS_2_ plates, which induce the vertical structure growth. As shown in Fig. 3c,d, when a high barrier of MoS_2_ islands or plates block the extension of MoS_2_ islands, the MoS_2_ islands may slip and change to vertical growth. Alternatively, when two MoS_2_ island push and collide against each other, the compression force may produce obvious distortion that an arch structure could be formed to release the pressure (Fig. 3g,h), which would subsequently act as the growth templet for the vertical growth of MoS_2_ nanosheets. Because vertical MoS_2_ are associated with MoS_2_ islands, it could be inferred that the alignment of vertically standing MoS_2_ nanosheets would be improved by synthesizing unanimous MoS_2_ islands with constant growth direction. MoS_2_ growth orientation has been reported to be facet-dependent^35^. Thus, the alignment might be improved by choosing a specific substrate.

**Figure 4:**
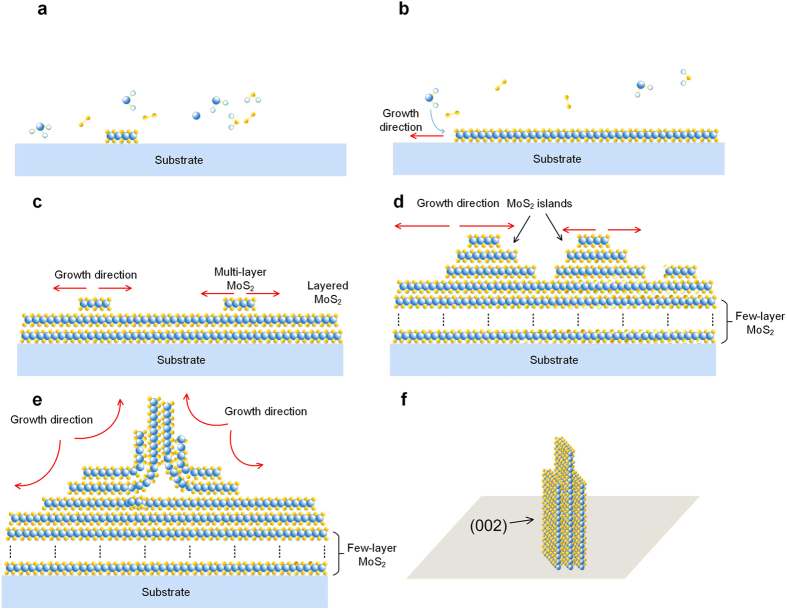
Growth model of vertically standing MoS_2_ nansheets. **(a)** “Nucleation stage”: nucleation sites were formed due to the reaction of MoO_3−X_ and S. (**b)** Two-dimensional MoS_2_ film was generally formed due to the growth and merge of seeds and bilayer MoS_2_ sheets were formed due to the continuous supply of reactant. (**c)** Multi-layer MoS_2_ films were generally formed. (**d)** MoS_2_ islands were formed beyond a certain critical MoS_2_ layers and their interaction may cause the accumulation of deformation energy. (**e)** The vertically standing MoS_2_ nanosheets may formed due to the horizontal and vertical compression and dilatation caused by the regional compression. (**f)** The simplified model for “vertical MoS_2_ nanobelts”.

To better understand the growth model, schematic graphs are shown in Fig. 4a–e. During the growth period, firstly, MoO_3_ powder was partially reduced by sulfur vapor to form volatile MoO_3−x_ or gaseous MoS_2_ and these sub-oxide compounds or gaseous MoS_2_ were adsorbed and diffused to the substrate, and subsequently formed nucleation sites of MoS_2_ films (Fig. 4a)^36^. With growth time increased, separated 2D MoS_2_ films were generally formed due to the growth and merging of the seeds as illustrated in Fig. 4b. It has been shown that the concentration of the gaseous MoS_2_ or sub-oxide compounds is an important thermodynamics and kinetics factor for the MoS_2_ growth^37^. Due to the high concentration of the reactants introduced by the fast evaporation of sulfur powder in our experiment (see Methods), the growth of MoS_2_ films would be facilitated by the supersaturated MoS_2_ vapor and sub-oxide compounds vapor. And multi-layer MoS_2_ films were consequently formed with increasing growth time. After that, MoS_2_ films are grown in a layer-by-layer fashion until a certain critical thickness (Fig. 4c). And then MoS_2_ islands were formed (Fig. 4d) to get an energetic favorable morphology according to the Stranski-Krastanov (SK) growth model^38^,^39^. With growth continued, MoS_2_ islands quickly extended and merged. A high strain energy would be introduced due to the fast chemical reaction. Thus, MoS_2_ seedlings could originate from the collision or distortion area of different MoS_2_ islands as shown in Fig. 4e. It is worth to note that though vertically standing MoS_2_ could originate from different based structures, the growth direction and the dominated surface facets remain the same: with 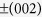 planes defining the two dominant surfaces (see Fig. 4f). The observed morphologies of the MoS_2_ are driven by the requirement for reducing surface energy. Such a {002}-dominant surface structure is caused by the lowest surface-energy of (002) surface, which is consistent with previous report^40^.

### HER and field emission performance

**Figure 5:**
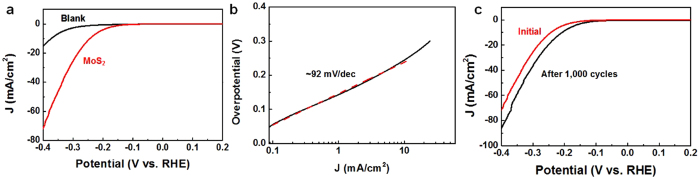
Electrochemical characterization of vertically standing MoS_2_ nanosheets. (**a,b)** Polarization curves and corresponding Tafel plots of as-grown vertically standing MoS_2_ films on Au foils. (**c)** Durability test for the as-grown sample.

To evaluate the catalytic activity of vertically standing MoS_2_ nanosheets, a typical three-electrode setup was used for HER test. Vertically standing MoS_2_ nanosheets were deposited on gold films using the same CVD method as described previously (see Supplementary Fig. S7 online). Typical cathodic polarization curves and corresponding Tafel plots are shown in Fig. 5a,b. The polarization curve of bare gold electrodes is also given in Fig. 5a. As it is known, Tafel slope is determined by the rate-limiting step of HER^41^. The Tafel slope in our sample was measured to be about 92 mV/decade (Fig. 5b). Previous studies have shown a large range of Tafel slope from 40 mV to 140 mV/dec and the reaction mechanism on MoS_2_ still remains inconclusive^18^,^19^,^20^,^21^,^42^,^43^.

The charge-transfer resistance could be estimated by using electrochemical impedance spectroscopy (EIS) method, revealing a lower charge-transfer resistance than gold films (see Supplementary Fig. S8 online). The exchange current density, j_0_, is determined by fitting the linear portion of Tafel plot at low cathodic current to the Tafel equation (see Supplementary Fig. S9 online). Based on the results, it is found the exchange current density j_0_ is about 22.3 μA/cm^2^, which is 70 times of bulk MoS_2_^44^. The large value of j_0_ is due to the high density of exposed edges^20^,^21^. However, the interlayer hopping of electrons between different MoS_2_ layers may limit the HER performance of vertically aligned MoS_2_ nanosheets^42^,^45^. Thus we believe the overall performance of our samples could be further improved by introducing doping during CVD process, which will be explored in the future. Another important criterion for a good electrocatalyst is its high durability. To evaluate this, continuous cyclic voltammograms were performed. The polarization curves before and after 1000 cycle are shown in Fig. 5c, where negligible loss of cathodic current could be observed.

**Figure 6:**
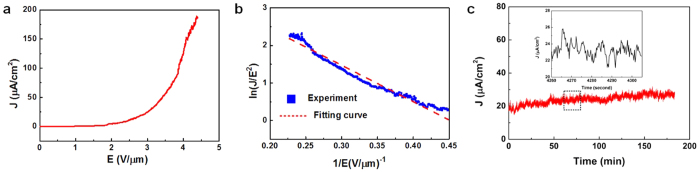
Field emission performance of vertically standing MoS_2_ nanosheets. (**a)** Electron field emission current as a function of applied electric field. (**b)** The corresponding Fowler-Nordheim (ln(*J*/*E*^2^) vs 1/*E*) plot. (**c)** Field emission current stability curves of the MoS_2_ nanosheets. The inset shows the change of current density in detail. The sampling interval is 200 ms in both picture.

In order to characterize the field-emission properties of vertically standing MoS_2_ nanosheets, a series of field emission measurement experiments were performed (Fig. 6). The anode was an indium tin oxide (ITO)-coated glass, and the vertically standing MoS_2_ nanosheets grown on SiO_2_/Si substrate was used as the cathode. Figure 6a shows the field-emission current versus electric field of the as-prepared MoS_2_ nanosheets. The turn-on electric field (J = 10 μA/cm^2^) is around 2.46 V/μm, which is smaller than previous reported MoS_2_ nanosheets (~2.8–5.5 V/μm)^25^,^46^,^47^. Fowler-Nordheim (FN) theory is the most commonly used model for understanding the electron-emission behaviour of various nanostructures. A modified F-N equation is used here to analyze the field emission property of MoS_2_, which could be expressed as:


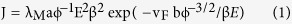


Where a = 1.54 × 10^−6^ A eV V^−1^ is a constant which depends on the surface structure and b = 6.83 × 10^7^ V cm^−1^ eV^−3/2^, J is the emission current density, E is the applied average field, ϕ is the work function of emitter, λ_M_ is the macroscopic pre-exponential correction factor and v_F_ (correction factor) is a particular value of the principal Schottky-Nordheim barrier function v^48^. The ratio of the actual local electric field and the applied average electric field is known as the field enhancement factor β. Fig. 6b shows the ln(J/E^2^) versus 1/E curve which has good agreement with the FN equation. The field-enhancement factor β of the vertical standing MoS_2_ sheets was calculated to be 6240 by taking the work function ϕ of bulk MoS_2_ to be 4.3 eV^49^. The large enhancement factor is due to the nanometric protrusions and sharp edges as we observed in Fig. 1.

The stability of emission current from the vertically standing MoS_2_ nanosheets was also measured (Fig. 6c). A stable emission current density of about 22 μA/cm^2^ over 170 min was recorded without any indication of degradation. Some spike like fluctuations were observed. The main reason of these fluctuations are believed to be caused by the adsorption/desorption and ion bombardment of residual molecules during the high field^50^. This test shows fairly stable emission current from vertically standing MoS_2_ nanosheets. The SEM observations on MoS_2_ nanosheets after field emission were carried out and shown in Supplementary Fig. S10. No severe deterioration of emitter surface was observed, demonstrating the stability of MoS_2_ nanosheets during field emission process.

## Discussion

We have developed a CVD process for synthesizing vertically standing MoS_2_ nanosheets. High density MoS_2_ nanosheets with sharp edges could be synthesized. TEM observations on the nucleation sites reveal the growth mechanism for the based structure of vertically standing MoS_2_. The based structures act as the growth templet and promote the subsequently vertical growth of MoS_2_ nanosheets. It is suggested that the high strain energy caused by the compression between MoS_2_ islands are the main reason for vertically standing MoS_2_ nanosheets growth. These MoS_2_ nanosheets exhibit enhanced field-emission properties with low turn-on electric field and good emission stability, suggesting promising in field emission based devices applications. In addition, we further confirmed the catalytic activity in HER. A high exchange current density of ~28 μA/cm^2^ is achieved, which is caused by high density of the exposed edge sites. More generally, the ultrathin material, that is, the three-atoms-thick MoS_2_, together with its vertical morphology, would hold great promising potential in catalytic, sensor, field emission applications.

## Methods

### Growth method

The MoS_2_ growth was performed in a conventional quartz tube. Silicon substrates with 285 nm SiO_2_ layer were cleaned in Piranha solution, followed by acetone, isopropanol and deionized bath for 5 minutes, and then finally dried using nitrogen gas. After that, samples were mounted on top of a quartz boat and faced down above high purity MoO_3_ powder (14 mg, 99.998%, Alfa Aesar). Also, 120 mg sulfur powder (99.5%, Alfa Aesar) was placed in a separate quartz boat located in the upstream of the quartz tube. The distance between sulfur powder and MoO_3_ powder was kept at 13cm. After that, the tube was pumped down to base pressure (<0.1Pa) and flushed with high purity nitrogen repeatedly. The tube was then filled with 1000 sccm nitrogen until one-atmosphere. During the synthesis process, the MoO_3_ was heated up to 750 °C at a rate of 15 °C/min in an argon environment at atmospheric pressure for 10 min. 5 sccm N_2_ was used as a carrying gas. Meanwhile, the sulfur was sublimated rapidly at approximately temperature of 700 °C. After growth procedure, the substrate was cooled down rapidly. Supplementary Fig. S11 shows the schematic diagram of the CVD equipment used in this study.

### Characterization

The surface morphology which reveals the coverage and uniformity of the grown MoS_2_ nanosheets was observed directly by SEM (Quanta FEG 450). Raman spectroscopy (Horiba, LabRAM HR-800), atomic force microscopy (AFM, Vecco Nanoscope IIIa) and transmission electron microscopy (TEM, FEI Tecnai G2 F20) were used to further characterize structure and quality of the as-grown MoS_2_ nanosheets.

A diode setup in a vacuum chamber was adopted for field emission tests. The prepared samples were placed as the cathode and an indium tin oxide (ITO)-coated glass was used as the anode. Five 150-μm-thick and electrically insulating alumina films were used as spacers, making the distance between the cathode and anode at 250 μm (see Supplementary Fig. S12 online). The base pressure of the vacuum chamber was kept at 1 × 10^−4^ Pa. The emission current versus the applied voltage were characterized automatically by a Keithley 2410 sourcemeter and a high voltage DC power supply. The field emission current stability was investigated using a computer controlled data acquisition system.

In order to evaluate the catalytic effects of those vertically standing MoS_2_ nanosheets, the HER tests were carried out. All of the electrochemical measurements were performed in 0.5 M H_2_SO_4_ solution using a three-electrode steup on an electrochemical workstation, with a saturated calomel electrode as the reference electrode (SCE), MoS_2_ nanosheets grown on Au films the working electrode and a Pt foil the counter electrode. It was calibrated with respect to reversible hydrogen electrode (RHE). The calibration was performed in the high purity H_2_ saturated electrolyte with two Pt foils as the working electrode and counter electrode. In 0.5 M H_2_SO_4_, E (RHE) = E (SCE) + 0.252 V. All the potentials reported in our manuscript are against RHE. Linear sweep voltammetry was conducted with a scan rate of 5 mV/s. And AC impedance measurement was carried out at an overpotential (η) of 0.12V with an AC voltage of 5 mV.

## Additional Information

**How to cite this article**: Li, H. *et al*. Synthesis and characterization of vertically standing MoS_2_ nanosheets. *Sci. Rep*. **6**, 21171; doi: 10.1038/srep21171 (2016).

## Supplementary Material

Supplementary Information
