# Supplementary material for: Synthesis and characterization of vertically standing MoS2 nanosheets
Source: Sci Rep. 2016 Feb 18;6:21171. doi: 10.1038/srep21171 (PMC4758069; doi:10.1038/srep21171)
Supplement: Supplementary Information [file srep21171-s1.doc]

**Supporting Information**

**Synthesis and characterization of vertically standing MoS_2_ nanosheets**

Han Li,^1^ Huaqiang Wu,*^1,2^ Shuoguo Yuan,^3^ and He Qian^1,2^

^1^Institute of Microelectronics, Tsinghua University, Beijing, China

^2^Tsinghua National Laboratory for Information Science and Technology (TNList), Beijing, China

^3^Department of Applied Physics, The Hong Kong Polytechnic University, Hong Kong, China

Corresponding Author

*Email: [wuhq@tsinghua.edu.cn](mailto:wuhq@tsinghua.edu.cn)

Table of content

Fig. S1: High resolution SEM image of MoS_2_ nanosheets.

Fig. S2: TEM observation of vertically standing MoS_2_ nanosheets.

Fig. S3: XPS spectrum scan.

Fig. S4: Raman spectrum of pristine and CVD MoS_2_ with different growth time.

Fig. S5: TEM image of intermediate state of vertically erect MoS_2_.

Fig. S6: EDX scan.

Fig. S7: SEM image of vertically standing MoS2 nanosheets grown on Au substrate.

Fig. S8: Nyquist plots of MoS_2_ and gold substrate.

Fig. S9: Tafel plot.

Fig. S10**:** SEM observations on MoS_2_ nanosheets before and after field emission test.

Fig. S11: Schematic view of the furnace setup

Fig. S12: Schematic of experimental setup of field emission test.


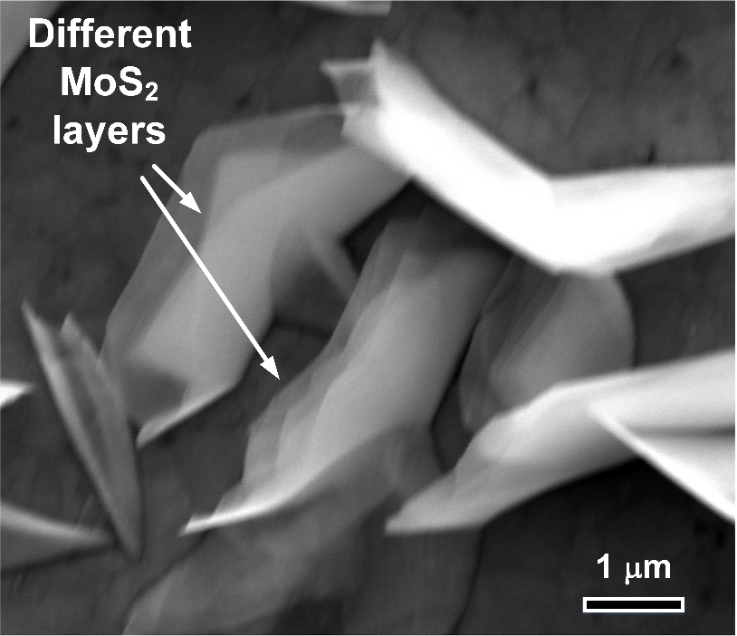


**Fig. S1** High resolution SEM image of MoS_2_ nanosheets. Edges associated with different MoS_2_ layers could clearly observed.


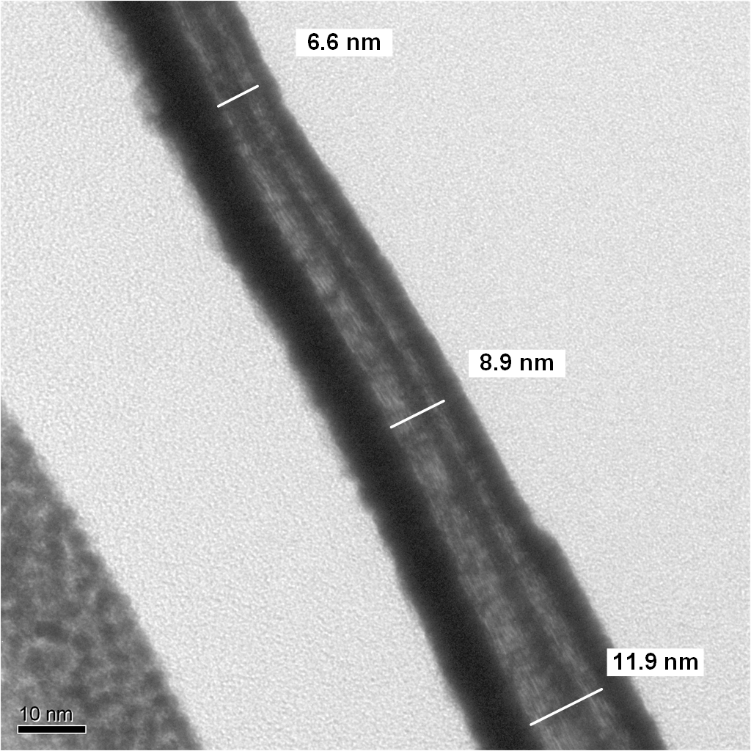


**Fig. S2** TEM observation on the middle region of vertically standing MoS_2_ nanosheets. The tapered structure of MoS_2_ nanosheets could be clearly observed. The thickness of MoS_2_ nanosheets are varied from 11.9nm to 8.9nm, and to 6.6nm from the root to the top in this image.


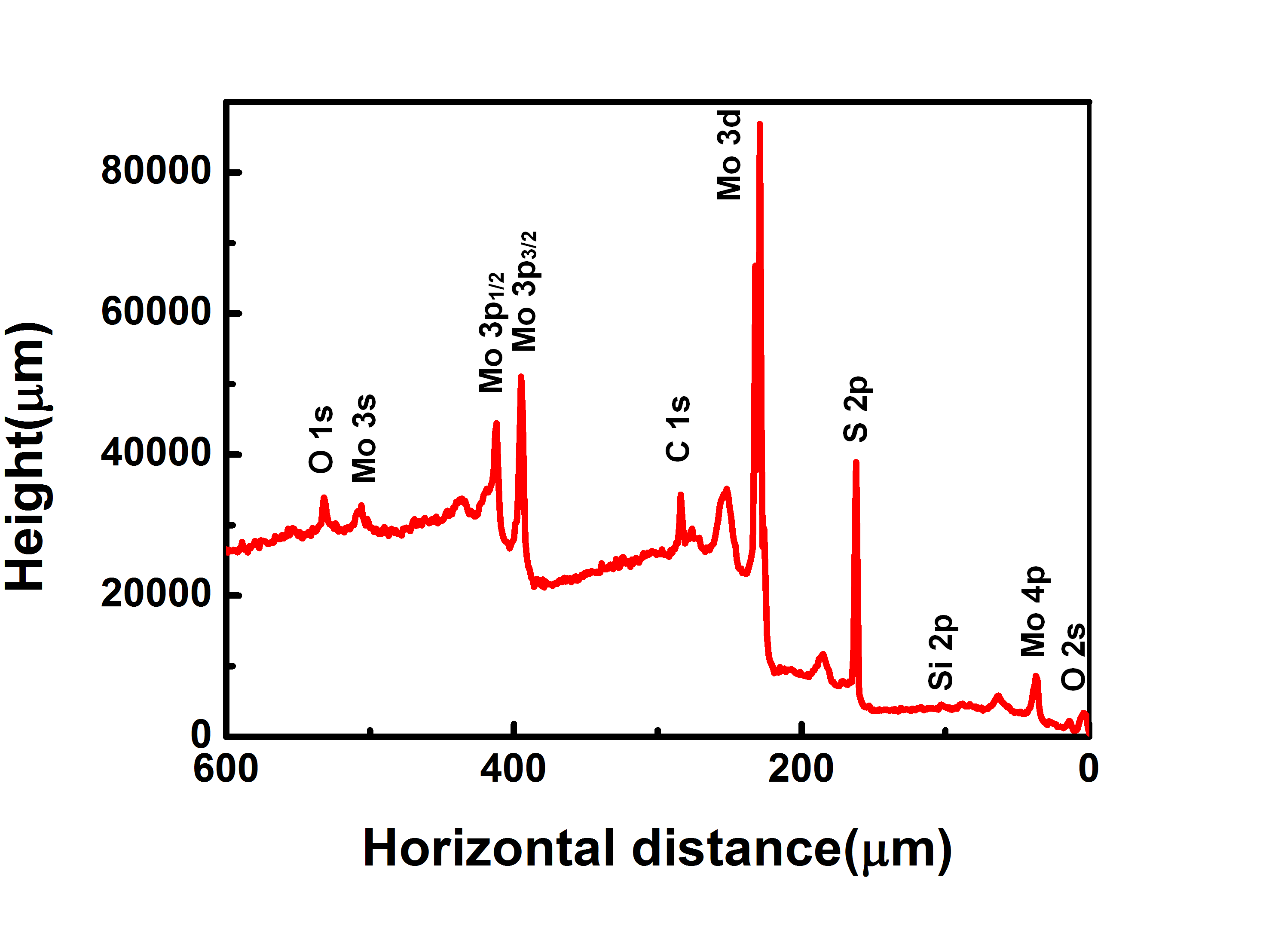


**Fig. S3** XPS spectrum scan of MoS_2_ samples grown by CVD. Two characteristic peaks of Mo 3d could be detected at 232.7 and 229.6 eV, attributed to the doublet Mo 3d_3/2_ and Mo 3d_5/2_ for Mo^+4^. The S 2p region also shows a doublet peak with the S 2p_1/2_ peak at 163.6 eV and S 2p_3/2_ peak at 162.4 eV.


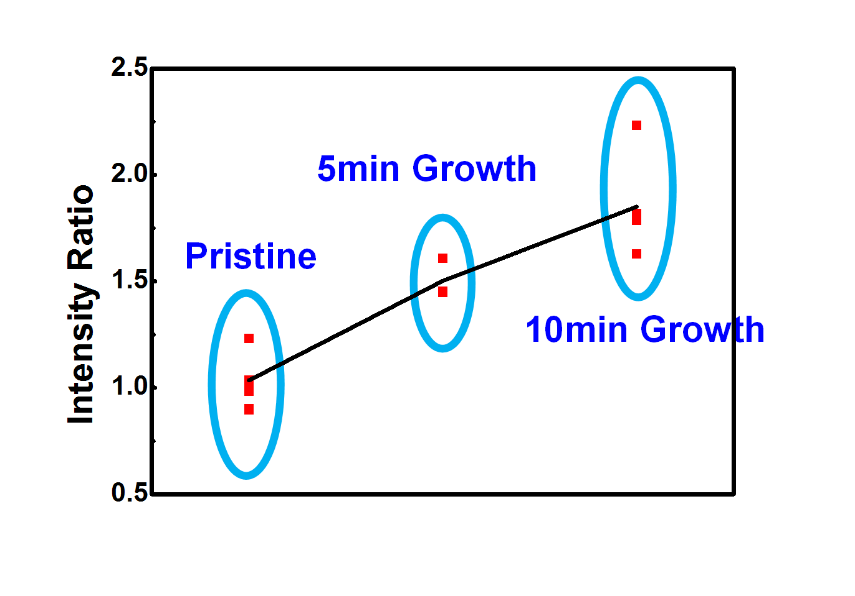


**Fig. S4** The normalized intensity ratio between A_1g_ and E^1^_2g_ of pristine MoS_2_ and our samples with different growth time. The higher ratio reveals the higher density of the exposed edges.


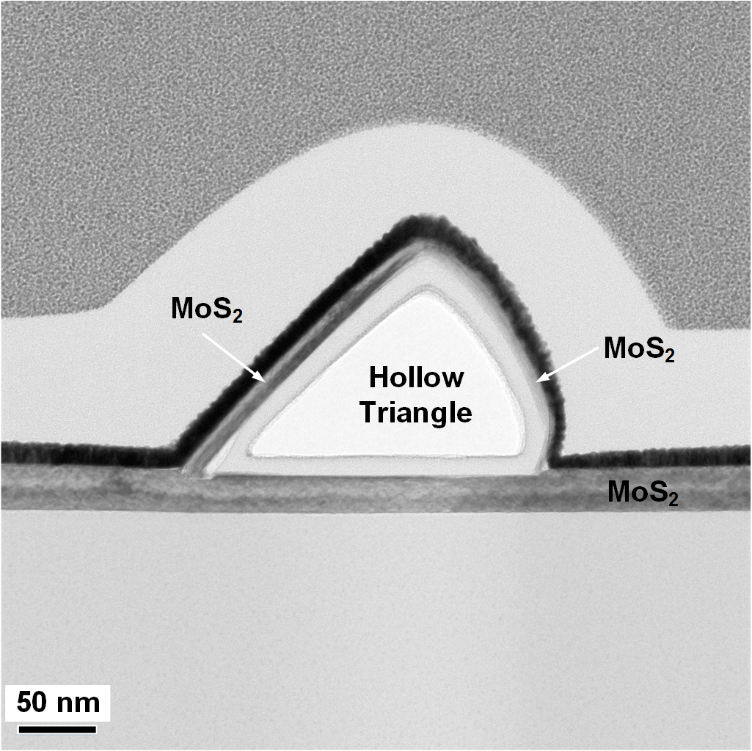


**Fig. S5** TEM image of intermediate state before the formation of vertically erect structure.


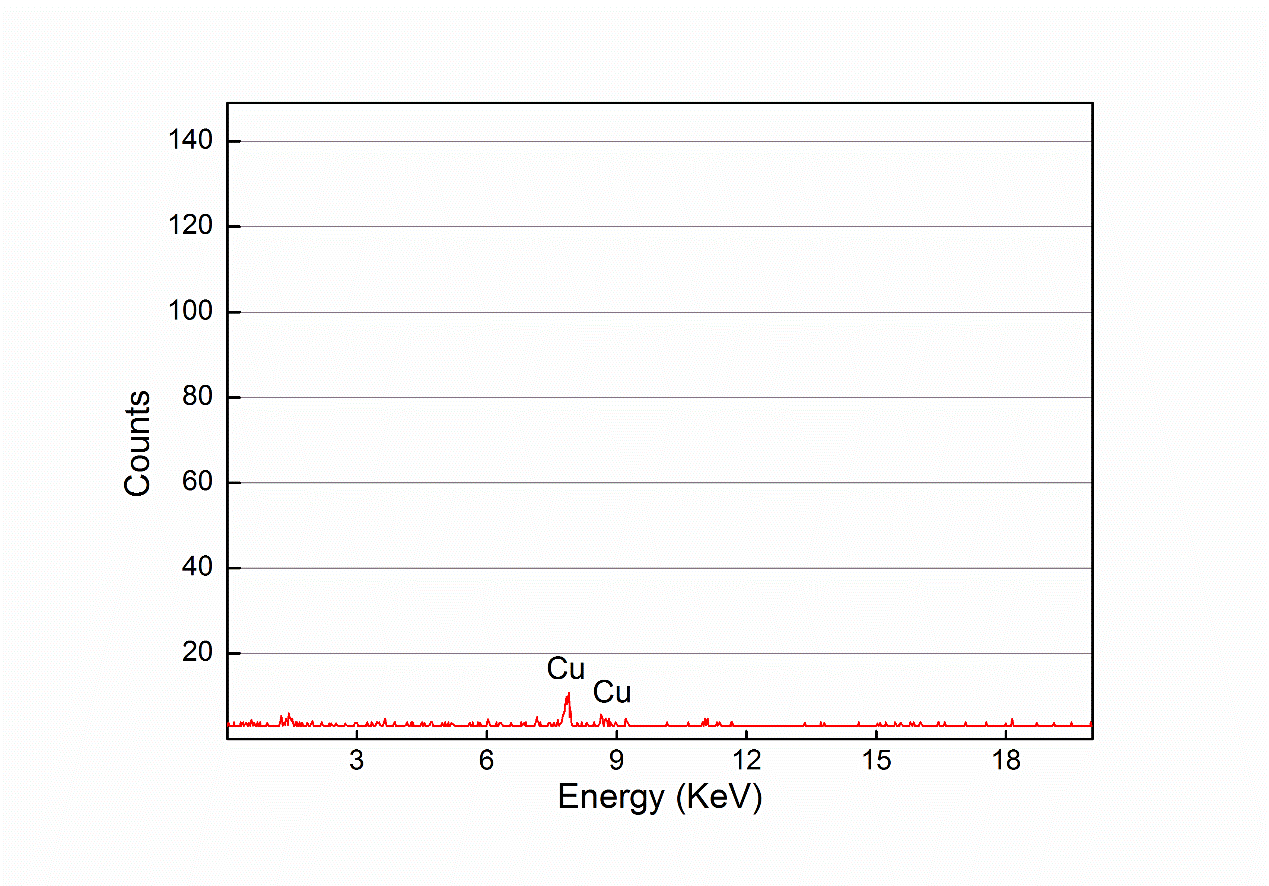
**Fig. S6** EDX scan on the triangle zone in Fig. S5. Cu signals came from TEM grid, which was used to support sample. No other elements were detected.


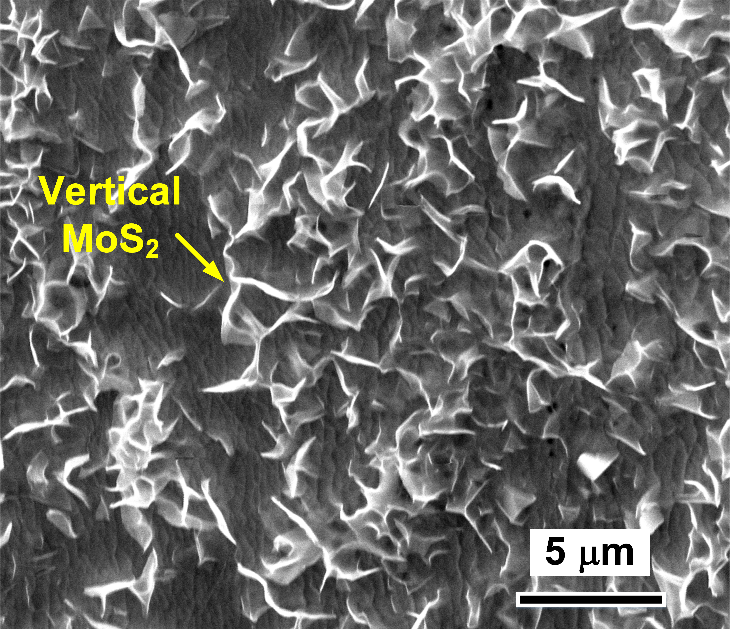


**Fig. S7** SEM image of vertically standing MoS2 nanosheets grown on Au substrate.


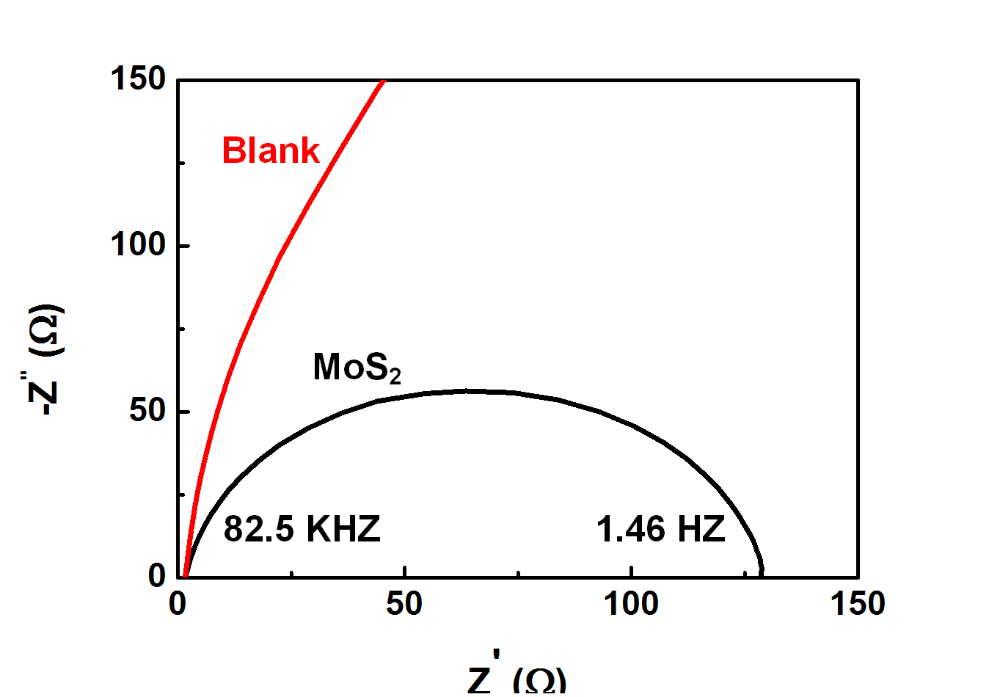


**Fig. S8** Nyquist plots of MoS_2_ and gold substrate. The Nyquist plots were obtained with frequencies ranging from100 kHz to 1Hz at an overpotential of 0.15 V.


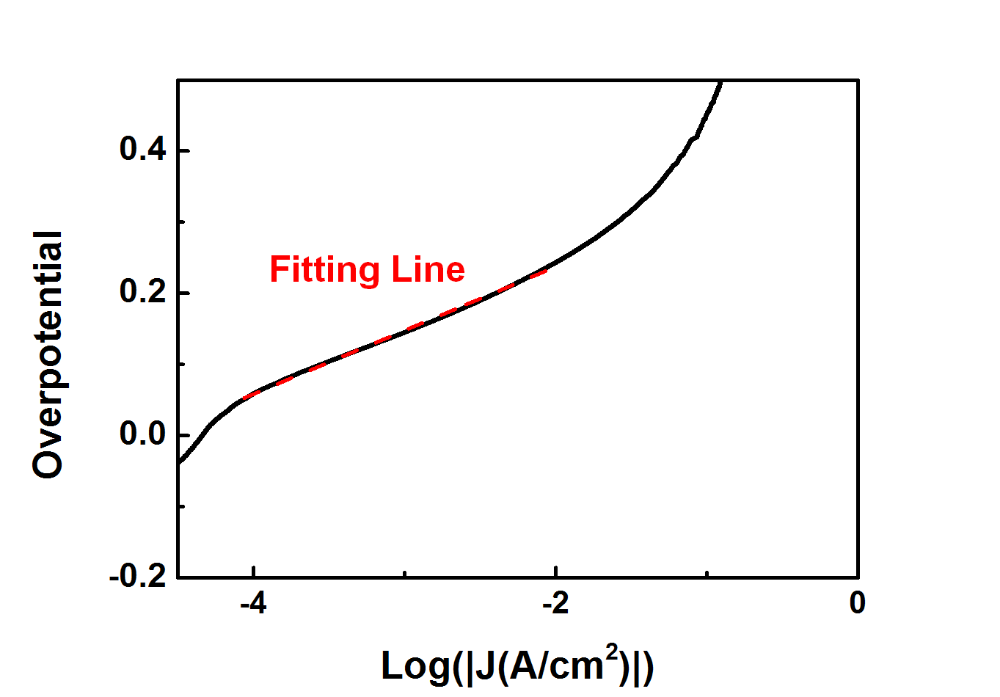


**Fig. S9** The exchange current density, j_0_, is determined by fitting the linear portion of Tafel plot at low cathodic current to the Tafel equation. The exchange current density could be obtained to be 22.3 μA/cm^2^ from the fitting line.


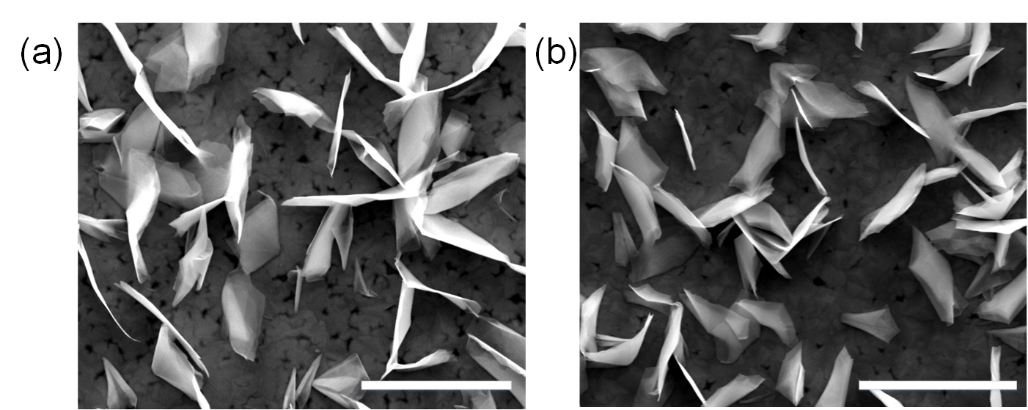


**Fig. S10** SEM observations on MoS_2_ nanosheets before (a) and after (b) field emission. No severe deterioration of emitter surface was observed


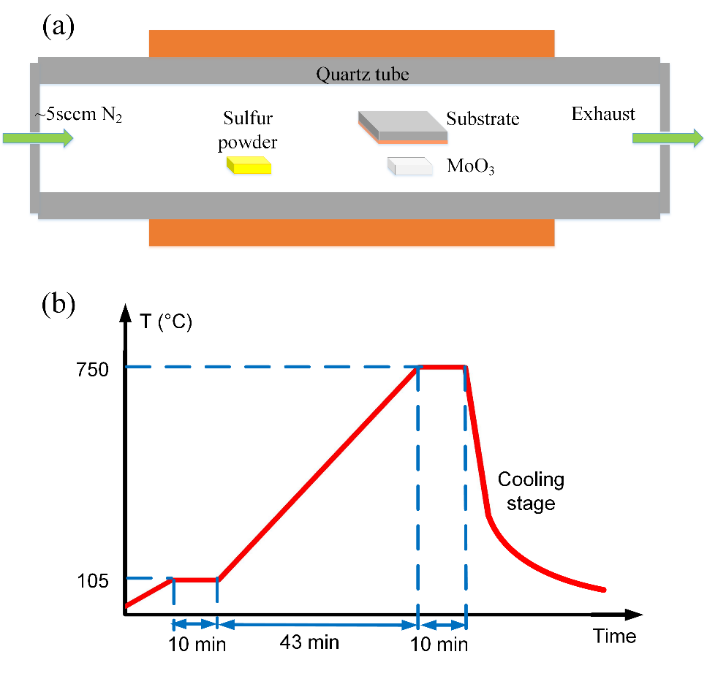


**Fig. S11** (a) Schematic view of the furnace setup during a typical MoS_2_ growth process. (b) Temperature profile during CVD process.


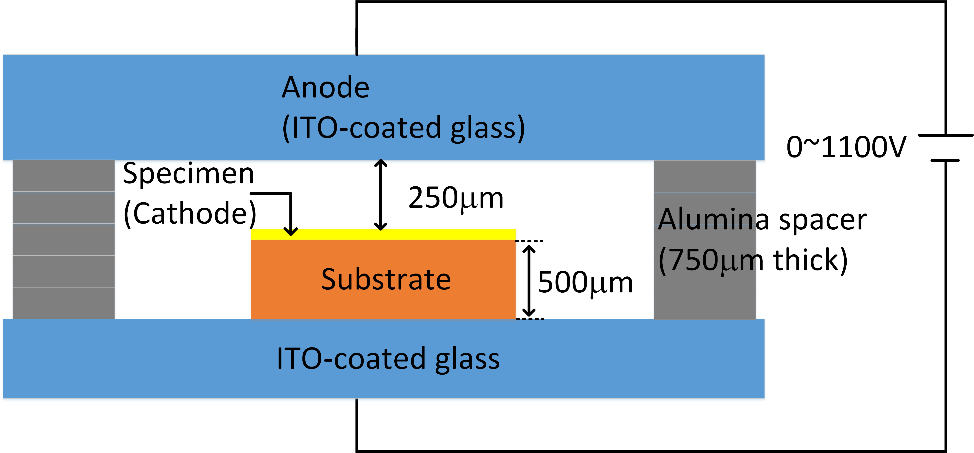


**Fig. S12** Schematic of experimental setup for field emission test. The prepared samples were placed as the cathode and an indium tin oxide (ITO)-coated glass was used as the anode. The emission current versus the applied voltage were characterized automatically by a Keithley 2410 sourcemeter and a high voltage DC power supply.
